# Renal Agenesis with Full Length Ipsilateral Refluxing Ureter

**Published:** 2016-04-24

**Authors:** Dilip Kumar Pal, Vipin Chandra, Manju Banerjee

**Affiliations:** 1 Department of Urology, Institute of Post Graduate Medical Education and Research, Kolkata-700020; 2 Department of Surgery, Institute of Post Graduate Medical Education and Research, Kolkata-700020

**Keywords:** Unilateral renal agenesis, Vesicoureteric reflux, Hematuria

## Abstract

Unilateral renal agenesis with vesicoureteral reflux in the ipsilateral full length ureter is a rare phenomenon. Herein we report a case of 10-year old boy who presented with recurrent urinary tract infections. No renal tissue was identified on left side in various imaging studies. Micturating cystourethrogram (MCUG) showed left sided refluxing and blind ending ureter. Left ureterectomy was done because of recurrent UTI in the refluxing system.

## CASE REPORT

A 10-year-old male child presented with several episodes of hematuria associated with mild dysuria for the last two years. He was a proven case of urinary tract infections (UTI) and was on chemoprophylaxis with nitrofurantoin (50 mg/day) since last 6 months. General physical and systemic examinations were normal. Urine examination revealed pus cells but culture grew no organisms. Hemogram, blood urea and serum creatinine levels were in normal range. Ultrasonography (USG) did not show left kidney in lumbar and other ectopic locations. Right kidney was normal in shape, size and location with normal echotexture. No evidence of dilated ureter on either side was reported. CECT with CT urogram confirmed the USG findings (Fig. 1). MCUG showed refluxing full length blind ending ureter on left side. Left ureter was minimally dilated without any tortuosity. Urinary bladder was normal (Fig. 2). DMSA scan showed no functional left renal tissue. Right kidney was normal on radioisotope scan. The diagnosis of left renal agenesis with blind ending left ureter and ipsilateral vesicoureteric reflux (VUR) was made. Antibiotics were given empirically for two weeks. MCUG, repeated after resolution of clinical symptoms and normalization of urine report, showed left sided VUR. Cystoscopy showed normal lower tract including a normal trigone. Retrograde pyelography (RGP) showed full length left ureter with proximal blind end. Due to recurrent UTI with VUR in spite of chemoprophylaxis, exploration with excision of the whole ureter was undertaken. On exploration no evidence of left renal tissue was found. Postoperative period was smooth and the child is symptom free at follow-up.

**Figure:**
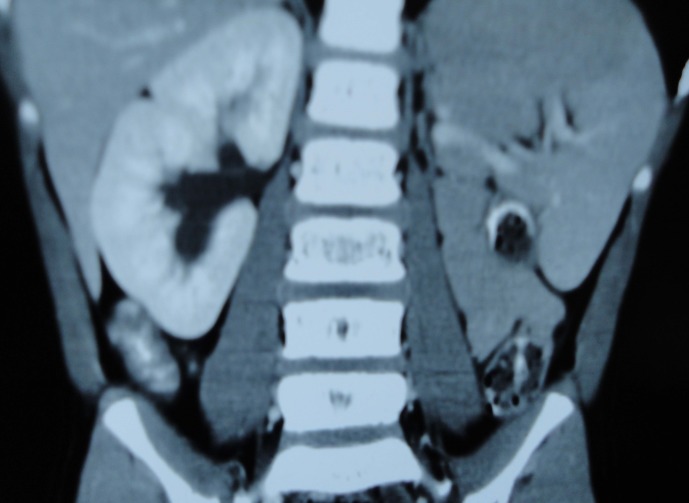
Figure 1:CECT of KUB region showing absent left kidney.

**Figure:**
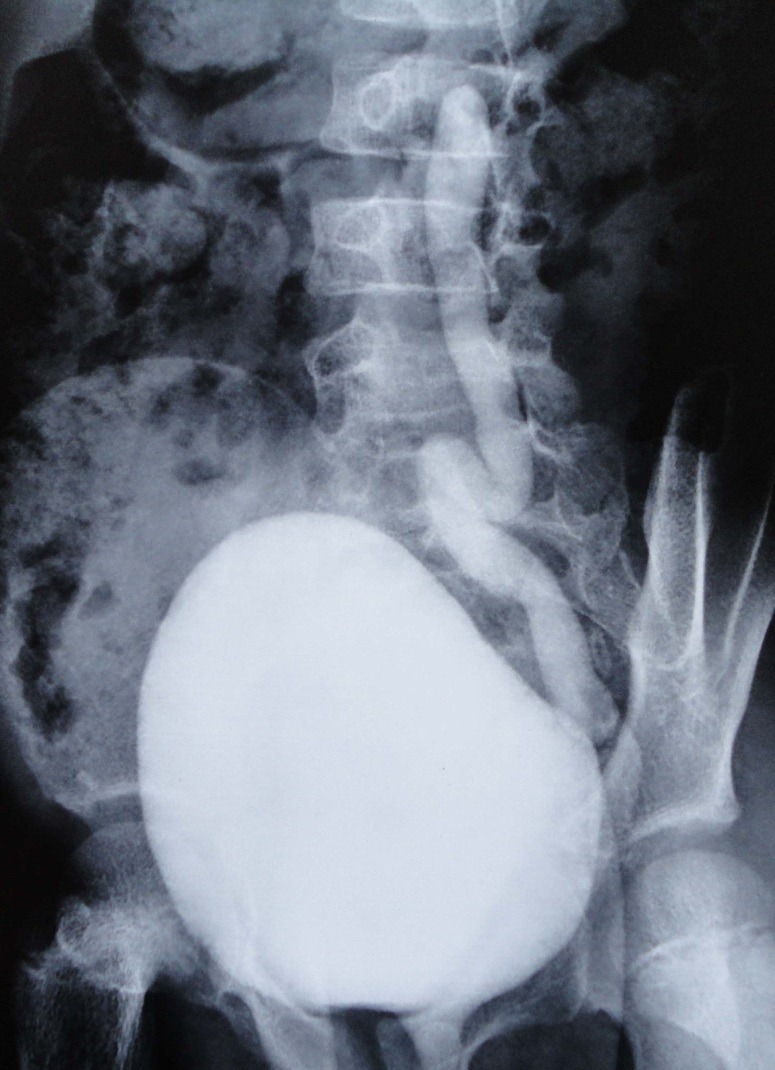
Figure 2:MCUG showing reflux in the whole length of left ureter with a normal bladder.

## DISCUSSION

Unilateral renal agenesis (URA) is an uncommon condition. Most cases of URA are associated with absent ipsilateral ureter or if ureter is present it is small near bladder end. Normally ureter develops from a bud which arises from mesonephric duct. Kidney develops when there is interaction between ureteric bud and metanephric blastema. In renal agenesis either ureteric bud is absent or interaction of ureteric bud with metanephric blastema does not occur.[1] When ureteric bud fails to fuse with renal plate, it results in blind ending ureter.[2] URA may be due to in-utero regression of multidysplastic kidney.[3]

Isolated URA is usually asymptomatic. A blind-ending ureteric bud has a higher than normal incidence of vesicoureteric reflux and therefore may get acutely inflamed. It may result in hematuria, calculus formation or recurrent UTI.[1] Systemic examination and routine urine examination may be normal as found in the index case.

MCUG is the gold standard to demonstrate the presence of VUR. Length of blind ending ureter and grade of reflux can also be determined. In our case, MCUG showed full length refluxing left sided blind ending ureter. Management of these cases depends upon symptoms. Surgical excision of blind ending ureter is advised in case of non-resolving VUR or breakthrough UTI.[4, 5] Both open and laparoscopic retroperitoneal approach may be used.. [6] Unilateral renal agenesis may be associated with ipsilateral refluxing blind ending full length ureter which can lead to recurrent urinary tract infection and its removal is thus indicated.

## Footnotes

**Source of Support:** Nil

**Conflict of Interest:** None declared

